# Communicating with graduate medical trainees: the Cleveland Clinic experience

**DOI:** 10.1007/s40037-013-0062-3

**Published:** 2013-06-05

**Authors:** Laura R. Greenwald, James K. Stoller

**Affiliations:** Education Institute, Cleveland Clinic, 9500 Euclid Avenue/NA22, Cleveland, OH 44195 USA

**Keywords:** Communication, Educational technology, Health information technology, Medical education, Post-graduate education, Medical student and resident education

## Abstract

Optimizing communication with graduate medical trainees is critical, as they contribute importantly to the mission of academic medical centres. Yet, communication is challenged by their complex schedules, geographic separation, and time constraints. Few studies have examined this issue to offer valuable solutions. Because traditional approaches are suboptimal, two communication tools were implemented: (1) a web-based intranet site called [graduate medical education] GME|com, and (2) an electronic newsletter, GME|com Headlines. The goals were to: (1) build a single repository of information relevant to trainees, programme directors, and coordinators, and (2) minimize their email burdens. A post-launch survey showed that >75 % of respondents indicated they visited the site and, of those, >90 % perceived value to the site. Analysis of use over the first year showed 39,377 visits (mean 108/day) and 93,785 pageviews. Sixty percent of users visited GME|com between 9 and 201 times and 18 % >201 times. A survey of programme directors from the 25 largest training programmes in the US confirmed the challenges of communicating with trainees and suboptimal results of current solutions. GME|com and Headlines represent complementary communication tools that have been well-received and frequently used. Future opportunities include assessing the association of GME|com use with increments in quality and patient safety.

## Background

In the context that academic medical centres host large numbers of graduate medical trainees in multiple medical specialities who provide frontline clinical care, communicating with all trainees effectively regarding key information such as quality and patient safety is both critical and challenging. Communication challenges include: (1) lack of opportunities for all trainees to convene at the same time and place, given the variety of clinical schedules, (2) lack of dedicated computers, and (3) time and clinical demands, which require markedly concise and impactful communication. Despite the challenge and the importance of communicating with graduate medical education (GME) trainees, the issue has received little formal attention to date. Because Cleveland Clinic hosts ~1,100 clinical trainees, 68 programmes overseen by the Accreditation Council on Graduate Medical Education (ACGME), and ~100 additional programmes, it is not surprising that we have experienced these challenges of communicating with GME trainees and herein propose a strategy to enhance such communication.

The current paper describes the development of and early experience with two complementary communication tools: (1) GME|com (short for ‘GME communication’), and (2) a bimonthly e-newsletter called ‘GME|com Headlines.’ GME|com is an intranet-based site that was developed based on trainees’ preferences regarding an optimal communication resource. GME|com Headlines was developed to consolidate information that otherwise would be sent in multiple emails, thereby reducing the email burden and helping to avert email fatigue. Specific goals for creating these tools were: (1) to create a single repository of information (e.g., policies, patient safety and quality initiatives, on-call schedules, procedural announcements, social events, etc.) relevant to trainees, and to programme directors and coordinators, (2) to minimize the email burden on trainees, and (3) to provide a channel to receive trainees’ candid online feedback regarding issues presented in GME|com.

To assess initial acceptance of these online tools, we surveyed trainees and analyzed utilization of the site. We also surveyed GME programme directors outside of Cleveland Clinic to assess perceptions and practices of communicating with their trainees.

This paper reports the results of trainees’ baseline input regarding an ideal communication site, a separate survey of outside GME programme directors’ experience of GME communication challenges and solutions, the design of the GME|com site, data regarding the first year of using these tools, and results of a post-launch survey regarding trainees’ initial experience with GME|com.

## Methods

The study was deemed exempt by the Cleveland Clinic Institutional Review Board.

The creation, implementation, and assessment of the GME|com intranet site occurred in three phases: (1) before designing the site, focus groups and a survey of GME trainees were conducted to understand trainees’ preferences about how best to communicate with them, (2) the GME|com site was then designed based on this feedback and launched, and (3) patterns of utilization and trainees’ reactions to GME|com were assessed by a post-launch survey.

A fourth aspect of the study was to assess other programmes’ approaches to communicating with GME trainees, for which a telephone survey of outside GME programme directors of the 25 largest US GME programmes (according to the ACGME [1]) was conducted.

Online and phone surveys and focus groups were organized and conducted with the Cleveland Clinic Market Research Department. Results of online surveys were tallied using Vision Critical (Vancouver, BC, Canada); telephone surveys were transcribed by 24/7 Transcripts (Las Vegas, Nevada).

### Baseline assessment of trainees’ preferences for an optimal communication approach

Of the 1,075 trainees invited to complete the baseline online survey in 2010, 94 responded (9 %), and 8 participated in two focus groups. Respondents expressed a desire for an intranet site dedicated solely to them and regarded email as the most effective way to communicate despite feeling overloaded by email. Respondents stated that they paid closest attention to information related to their everyday work, e.g., information on patient safety updates, practice requirements, etc. Practical information was especially valued, e.g., where to get a new identification badge, how to obtain medical records, where to find details about upcoming examinations and events, and where to get information on license renewals. Also, respondents wished to receive information regarding events in a comprehensive calendar, classifieds, GME news, on-call schedules, job opportunities, and peer profiles. Notably, little interest—even some wariness—was expressed regarding offering social networking on GME|com.

### GME|com site design and implementation

Based on survey and focus group feedback, and with the full involvement and support of institutional GME programme directors and GME leadership, construction of the GME|com site began in November 2010 and the site was launched on 1 February 2011. The launch was announced to trainees in several ways:

1. Via direct email;
2. In announcements to all programme directors and coordinators with a request to share this information with trainees in their programmes;
3. Through the GME|com Headlines e-newsletter itself.

The site is administered by a communications specialist in the Cleveland Clinic Education Institute (LRG).

**Fig. 1:**
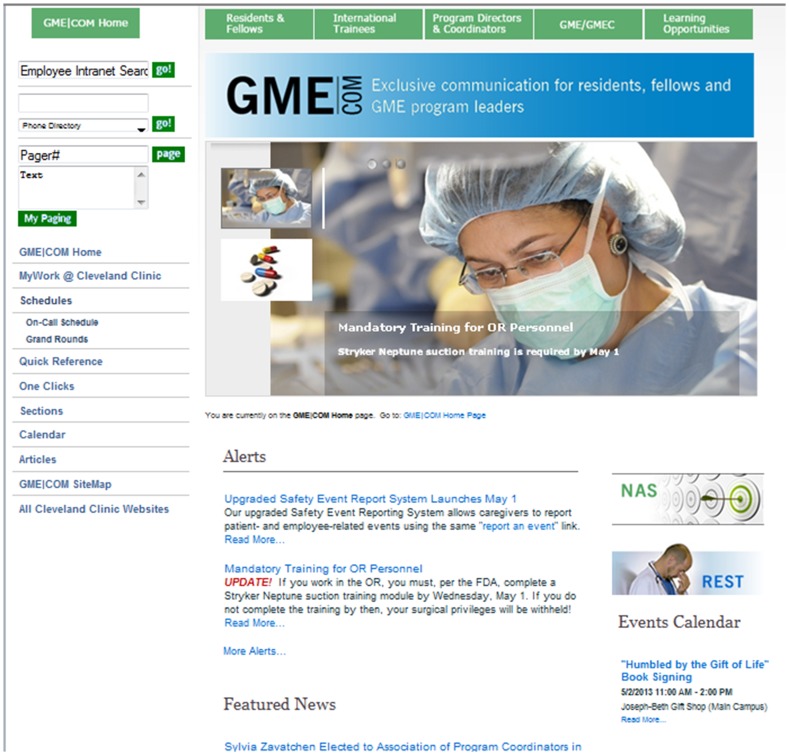
Screenshot of the GME|com intranet site

Figure 1 shows a screenshot of GME|com, for which the tagline ‘*Exclusive communication for residents*, *fellows and GME programme leaders*’ was developed. Trainees can access GME|com both inside and via remote access outside of Cleveland Clinic. Key features of GME|com include:

1. Access to utilities for trainees (e.g., pager and phone directory; local weather and time; on-call directory; employee services such as employee benefits, dining menus, product/service discounts, and volunteer opportunities; housing information; and one-click links to the electronic medical record log-in, the main Cleveland Clinic intranet site, House Staff Association (HSA) pages, and more). These utilities are placed on the left side of the site for easy access.
2. ‘Alerts,’ which comprise ‘need-to-know’ information for trainees (e.g., notices regarding safety and quality initiatives such as central line-associated bloodstream infection training, corporate compliance such as HIPAA issues, and clinical protocol alerts). These alerts are placed on the centre of the site, ‘above the fold’ for emphasis.
3. ‘News,’ which includes non-clinical information for trainees (e.g., organizational updates, recognition, and educational opportunities). News is located on the centre of the site beneath alerts.
4. Event information with a quick click to view the full calendar; clinical and quality updates; a link to the current issue of GME|com Headlines (Fig. 2); and a button to submit comments or suggestions. These links are placed along the right margin.

**Fig. 2:**
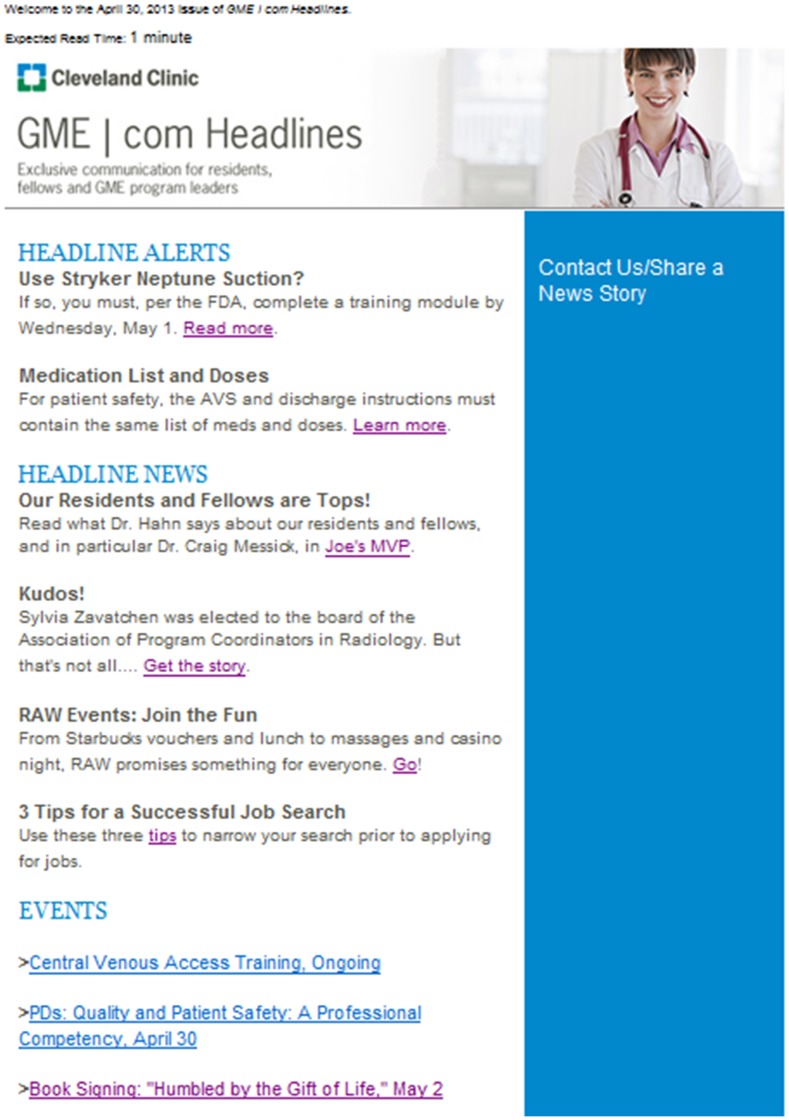
Screenshot of GME|com Headlines, a bimonthly electronic newsletter; the headlines contain ‘teasers’ that hotlink to full content regarding the issue
5. Profiles on trainees and GME staff (i.e., short bio and picture). These profiles are presented centrally, ‘below the fold.’
6. Career services (e.g., help with resume development and contract negotiation) through the Office of Physician Recruitment. This information is posted on the left side.
7. A designated link for Residents/Fellows, which takes them to a landing page that repeats the home page alerts as well as links to information on benefits and employment, the HSA, GME physicians’ manual, etc.
8. A designated link for international trainees, which takes them to pages with information on visas, maintaining status, international travel, and immigration resources.
9. A designated link for GME programme directors and coordinators, featuring announcements of policy changes, important date milestones, lectures and meetings, and a staff directory.
10. A Comment/Feedback button on every main landing page and on the home page to encourage feedback to be sent to a GME email account monitored by the site administrator.

The cost to build the site was low; most of the design and programming work was performed in-house. A rotating banner for the site, initial site build and an e-newsletter template were created by vendors, the total cost for which was approximately $3,500.

## Results

Before GME|com was launched, trainees relied on three websites for GME news and information: (1) the GME Centre’s intranet site, which had not been designed to serve as a main communication vehicle for trainees, (2) the HSA site, which primarily listed social and educational events, and (3) MedHub (MedHub, Ann Arbor, MI), a purchased site primarily used to gather and manage trainees’ teaching evaluations and to log duty hours.

GME|com was designed to offer new, desirable features for trainees based on the baseline feedback. Its implementation replaced both the GME Centre’s and the HSA intranet sites.

### Trainees’ early experience with GME|com

In 2011, six months after GME|com was launched, all clinical trainees (*N* = 1,130 at that time) were invited to complete an electronic survey regarding the frequency with which they visited GME|com, preferences and dislikes regarding the site and the e-newsletter, and suggestions for improvement. The survey invitation was emailed to all trainees directly from our Market Research Department, and trainees were assured that their responses would be kept anonymous.

Fifteen percent, or 175 trainees, responded. Most had visited the site and found it useful (Table 1). More than 80 % of respondents recalled receiving the e-newsletter, and most reported at least skimming the headlines. Importantly, regarding one of the main goals for GME|com, most respondents (>80 %) reported receiving fewer GME-related emails since the launch of the GME e-newsletter. The most common suggestions to enhance GME|com included adding clinical practice tips and making the GME staff directory easier to find.

**Table 1:** Survey results for GME|com

Recall/usage	>75 % have visited site
	Of those, >90 % report site as being at least somewhat useful
Most useful features (in decreasing order of priority)	Centralized source for GME information
	Relevant news/headlines
	Events/calendar
	Direct access to resources
	Contact information
Least useful feature	Feedback button
Suggested improvements	Adding board review practice questions
	Adding quick clinical practice tips
	Forum to chat/share ideas with other trainees
	Posting GME staff contact information in easy-to-find place
	Designating site as the home page when trainees log-in

**Table 2:** Survey results of outside GME programmes: pros and cons of communication strategies

Communications	Advantages	Disadvantages
Email	Trainees use email daily so they are likely to receive the message Sending messages via email is easy. Most programmes have an email list of trainees to use	Trainees receive a large volume of email and likely miss important information because of email fatigue Not all messages may be deemed important by trainees and they may ignore them Keeping the list of email addresses updated can be challenging for programmes with several locations and systems; email addresses change when trainees change locations
Cascading communication	Trainees are more likely to pay attention to messages that are sent by someone familiar	There can be uncertainty as to whether messages are being cascaded to trainees Leadership may be too busy to, forget to, or, for various reasons, may not be motivated to cascade the message

**Fig. 3:**
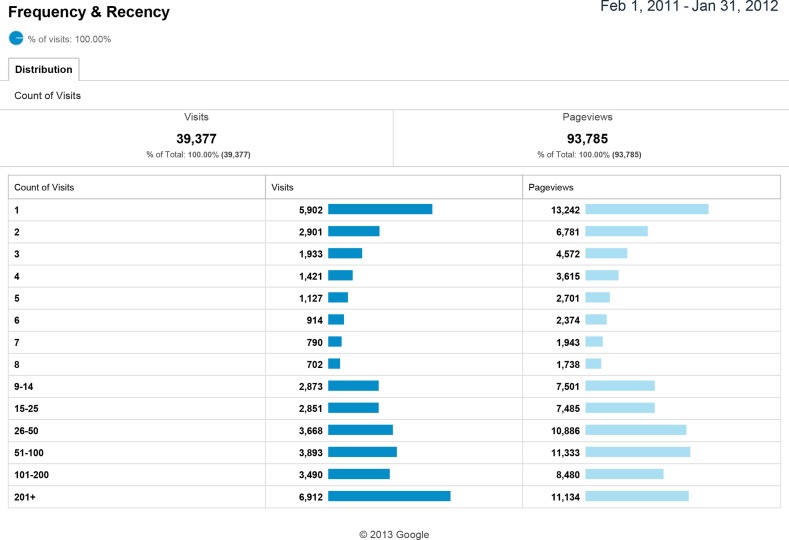
Google Analytics analysis of utilization of GME|com from 1 February 2011 to 31 January 2012

Regarding the frequency and patterns of site use, for the first year of GME|com use (2/1/11–1/31/12), 39,377 visits and 93,785 pageviews were recorded. Mean time spent on the site was 2 min 34 s. Each month, 15 % of users were new to the site, and 85 % were returning visitors (Fig. 3). Total visits to the Residents/Fellows tab within GME|com (Fig. 1) numbered 5,131. Descriptive statistics regarding use of GME|com were derived using Google Analytics (Google, Mountain View, Calif.).

**Fig. 4:**
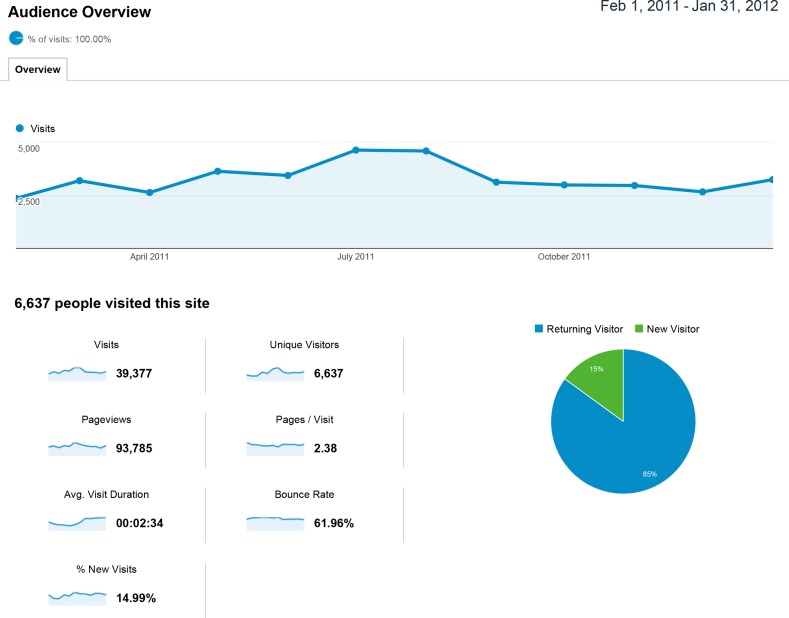
Google Analytics analysis of frequency of visits to GME|com from 1 February 2011 to 31 January 2012

Analysis of usage patterns showed upward trends in all categories, with consistent numbers of new users monthly and utilization spikes on the day the e-newsletter was sent. Specifically, the site averaged 108 visits per day and 257 pageviews per daily visit. Sixty percent of visitors had been to the site between 9 and 201 times, and 18 % of visitors had been to the site >201 times (Fig. 4). Since launch, the top-viewed content has shifted from corporate compliance articles (e.g., HIPAA issues) to alerts about GME policy (e.g., on-call meal eligibility) and clinical updates.

To further assess the impact and reach of GME|com, the frequency of pageviews of GME|com was compared with that of MedHub before the launch of GME|com for the same calendar interval in 2010–2011 (MedHub) and 2011–2012 (GME|com). The mean number of monthly pageviews of GME|com nearly tripled (33 vs. 12).

Since launch, two options to elicit feedback have been tried. The first, a feedback button that generated an email (and therefore not anonymous) was sparsely used. To encourage feedback, 1 year after launch, an anonymous comment feature was added. For a 1-year period (February 2012–January 2013), 95 comments were received (mean 8/month). Of those, 46 % of the comments were congratulatory or thankful in nature; 15 % related to employment benefits; 12 % related to practice or services; 11 % were suggestions; 7 % were complaints; and the remaining 9 % were miscellaneous in nature.

To capture trainees’ concerns about duty hour, fatigue and supervision issues, an anonymous online survey tool was added to GME|com at the beginning of 2012. To date, three concerns have been received, all of which prompted an immediate response from GME leadership.

### Survey of GME programme directors regarding their experience with trainee communication

Of the 25 directors of GME programmes invited to participate in a telephone survey, 11 responded (44 %). All respondents indicated that communicating with trainees was a high priority. On a Likert scale of 1–10, with 10 being the highest priority, 55 % rated the priority of trainee communication as 10; 9 % rated it as 9; and 36 % as 8.

While email was the most commonly cited method of communicating with trainees, 27 % of programmes did not report using email as a communication vehicle, relying mainly on cascading information to trainees (Box 1). Use of intranet sites was usually for posting policies rather than communicating information. Somewhat akin to GME|com, one programme reported using an internet site to communicate more broadly, i.e., to post resources, newsletters, policies, events, and other information for both current and prospective trainees. Unlike GME|com, however, this programme’s site did not use a ‘push’ strategy, i.e., sending GME|com Headlines, to entice trainees to visit the site regularly.

**Box 1:** Methods of communicating with GME trainees reported by GME programme directors

• Mass emails (varying degrees of frequency)
• Cascading communication
• Intranet (mainly as repository for policy documents and forms)
• Electronic newsletters
• Grand rounds/town hall meetings
• Residency management site (e.g., MedHub)

Many respondents expressed the need for multiple communication channels with trainees (Table 2). Most respondents also acknowledged the challenge of communicating with GME trainees, citing special challenges of geographic spread in programmes in which trainees served in multiple clinical locations, and lack of a centralized information technology function in programmes that functioned as a consortium of different hospitals and clinics. Several respondents had explored using social media for communication but ultimately rejected the idea over concerns regarding security and privacy.

## Discussion

This report introduces a web-based tool, GME|com, to address a challenge that our survey of GME directors indicates is widely shared.

Our main findings in assessing communication preferences of trainees and reactions to GME|com are that:

1. Trainees, programme directors, and GME leadership strongly endorsed the need for enhanced communication.
2. GME|com was designed based on input from trainees regarding their communication preferences and was well-received, with most trainees visiting the site, a loyal cohort of frequent users, and a steadily increasing number of new visitors.
3. Feedback regarding GME|com was generally very favourable, and most respondents (>80 %) reported receiving fewer GME-related emails since the launch of the GME e-newsletter.
4. Suggestions included adding clinical practice tips and making the GME staff directory easier to find.
5. Trainees have used the feedback mechanism mainly for inquiries regarding policy and benefit matters.
6. In contrast to the strategy behind GME|com and GME|com Headlines, which combine ‘push’ and ‘pull’ tactics, most of the large GME programmes that were surveyed use a ‘push’ strategy for communicating with trainees, i.e., mainly relying on email and cascading communication tactics.

This study extends available reports regarding communicating with GME trainees by describing GME|com as a dedicated, customized resource. To our knowledge, and based on a Medline search (1966–January 2012) using the search terms “education,” “medical,” “graduate,” “residency,” “computer communication networks,” and other communication terms, the use of web-based tools to communicate with GME trainees is unique, with few other available reports specifically addressing this topic [2–10]. Indeed, only three citations [2–4] addressed communicating with GME trainees directly, with most regarding recruitment, programme evaluation, or emergency communication. Among these three available reports, Fortin et al. [2] developed a successful internet-based tool for the Yale Primary Care Internal Medicine Residency Programme. Unlike GME|com, this site was devoted to one 74-resident programme, whereas GME|com serves Cleveland Clinic’s 165 training programmes and 15-fold more trainees. Also, unlike GME|com, the Yale site is internet-based and mainly features resident schedules as the main attractor (vs. GME|com Headlines).

In a second report, Zucker et al. [3] piloted an instructional intranet site for the Paediatrics Department at the University of South Florida College of Medicine. Unlike GME|com, the South Florida site was designed mainly as an instructional tool, hosting the complete paediatrics curriculum. The site was also used as a repository for documents, a directory of contact information, and a bulletin board with event information and resident birthdays, and less as a communication vehicle for clinical and quality news and updates.

Finally, Triola and Blaser [4] developed an email alert system for internal medicine physicians at New York University (NYU) School of Medicine, the goal of which was to effectively communicate emergent information to a diverse group of faculty and trainees. In contrast to the goal of GME|com to lessen the email burden to trainees, the NYU system was email-based.

In addition, we identified a Dutch internet site called Artsennet [11], which aggregates and summarizes news and medical information for healthcare providers. GME|com is distinguished from Artsennet and from other general healthcare sites by being a closed, non-commercial intranet-based resource that is customized for and directed to GME trainees rather than to a broader medical audience. Unlike Artsennet, the goal of GME|com is not to summarize recent medical findings and news but rather to disseminate information that is relevant to trainees’ training experience, e.g., schedules, policies, colleagues’ accomplishments, attention to quality metrics, etc.

We acknowledge the ubiquitous use of web-based tools in industries including healthcare. However, the paucity of available reports about communicating with GME trainees underscores the strength of GME|com as a strategy to engage and communicate with trainees.

Our survey of 11 other large GME programmes identified only a single programme using a web-based approach for comprehensive communication with trainees. That programme reported a single internet-based site that includes news and information for both current and prospective trainees. In contrast to GME|com, most (73 %) programme directors reported using email to communicate with residents, and many also rely heavily on cascading communication via programme directors, programme coordinators, and house staff leadership. This preference aligns with the suggestion from a Northwestern University study that email was the most effective communication tool for its internal audiences, emphasizing that ‘to be effective, information needs to be in a digital format and needs to be pushed to its intended audience’ [10]. That said, results of our baseline focus groups indicated that trainees felt burdened by the volume of their emails, prompting enthusiasm for a tool like GME|com that could lessen their email load.

Because GME|com is designed as a resource for trainees, programme directors, and coordinators, it aspires to a broader strategy that is more audience-inclusive and that combines both ‘push’ and ‘pull’ methods of communication. The bi-monthly mass email is sent (i.e., ‘pushed’ out) to trainees and to programme directors and coordinators and invites them to visit GME|com (i.e., ‘pulls’) to receive other nested relevant information on the site.

Based on baseline feedback from trainees suggesting email fatigue, GME|com was designed to obviate the need for frequent email communications to trainees from the GME Department and other Cleveland Clinic departments that often wish to communicate with trainees. Survey respondents confirmed this effect of GME|com. In the same spirit, with GME|com available, email access to the entire trainee email group is now limited by GME administration, with far fewer mass emails sent.

GME|com also differs in important ways from some of the strategies for communicating with other student and trainee groups that have been described (e.g., college undergraduates, etc.). Most reports on higher education communication focus on emergency communication, such as when a school shooting occurs, and include approaches such as blast emails, instant messaging, digital message boards, and mass phone call and text alerts [5–9]. GME|com is not designed to communicate emergency information; rather, it is a dedicated site that is updated daily with customized, focused content for its specific audience.

Several shortcomings of this analysis warrant comment. First, feedback regarding the design of GME|com and its use was available from only a minority of users, raising the possibility of response bias. At the same time, the wide utilization of data suggests that most Cleveland Clinic trainees have visited the site and that many users visit GME|com repeatedly and frequently, which testifies to the popularity of the site and to its responsiveness to trainees’ communication preferences.

Second, although we identified the total number of unique visitors to GME|com, the analytic tools precluded confidently identifying how many unique visitors were trainees versus members of other interested user communities (e.g., programme directors, coordinators). Still, because the site features different tabs for Residents/Fellows, programme directors and coordinators, etc. (Fig. 1), we suspect that the 5,131 pageviews of the Residents/Fellows landing page likely represent trainee visits. This number, which exceeds the total number of GME trainees yearly by 4.5-fold, supports the impression that the site is very widely used by trainees.

A third potential limitation is that although GME|com was designed to communicate content regarding important quality and patient safety information, the current study was not designed to assess the clinical impact or outcomes related to this information sharing. Indeed, temporal trends of increasing institutional quality metrics (e.g., HCAHPS scores, etc.) cannot be ascribed to GME|com solely, because many other healthcare providers (e.g., nurses, faculty, allied health providers, etc.) contribute importantly to these outcomes and because GME|com is not the only mechanism by which this information is communicated. Still, we believe that GME|com has contributed importantly to enhanced quality and clinical metrics over the time of its use.

Both GME|com and the feedback mechanisms on the website and in the e-newsletter are emphasized at the new trainees’ orientation. In order to limit the e-newsletter reading time to between 1 and 2 min, we have increased its frequency to once a week, usually early Tuesday morning so that trainees know when to expect it. Feedback on this increased frequency has been favourable.

In summary, we describe a customized intranet-based tool designed to engage and communicate with GME trainees regarding issues relevant to their training and to the clinical quality and patient safety missions of an academic medical centre (Cleveland Clinic). Early experience with GME|com has been very favourable. In the current quality and clinical outcomes-attentive climate, these analyses indicate future opportunities to extend the assessment of the site impact by identifying and reporting GME-specific metrics of patient safety and clinical quality.

## Essentials

- Communicating to graduate medical trainees is challenged by complex schedules, geographic separation and time constraints.
- Most other large GME programmes that were surveyed use a ‘push’ strategy for communicating with trainees, mainly relying on email and cascading communication tactics.
- Our experience shows that a dedicated intranet site and e-newsletter are efficient and cost-effective methods of communicating with a large, diverse employee population.
- Feedback on these communication initiatives has been favourable, with most Cleveland Clinic trainees visiting the site repeatedly and frequently.
- Future opportunities include assessing site usage with increments in quality and patient safety.

## References

[1] Accreditation Council for Graduate Medical Education. Data Resource Book. 2009–2010 Academic Year. Chicago: The Council; 2010.

[2] Fortin AH, Luzzi K, Galaty L, Wong JG, Huot SJ (2002). Developing an Internet-based communication system for residency training programmes. J Gen Intern Med.

[3] Zucker S, White JA, Fabri PJ, Khonsari LS (1998). Instructional intranets in graduate medical education. Acad Med.

[4] Triola MM, Blaser MJ. An email alert system for internal medicine physicians. AMIA annual symposium proceedings; 2003, p. 1035. PMC1480223 14728538

[5] Black JR (2009). Emerging trends. Am Sch Univ Mag.

[6] Butler AM, Lafreniere KD (2010). Campus reactions to mass notification. J Coll Stud Dev.

[7] Sander L (2008). At Northern Illinois U., leaders grapple with a tragedy. Chron High Educ.

[8] Schaffhauser D (2007). 7 Best practices for emergency notification. Campus Technol.

[9] Violino B (2008). Alert!: in emergencies, schools use technology to get the message out quickly. Community Coll J.

[10] Cubbage A (2009). Messages from within: communicating with internal audiences is increasingly important. Currents.

[11] www.artsennet.nl. Accessed 21 April 2013.

